# Supplementary material for: A Systematic Review of Sleep–Wake Disorder Diagnostic Criteria Reliability Studies
Source: Biomedicines. 2022 Jul 6;10(7):1616. doi: 10.3390/biomedicines10071616 (PMC9313077; doi:10.3390/biomedicines10071616)
Supplement: Supplementary file 1 [file biomedicines-10-01616-s001.zip › Supp. Mat/Table S1 (Search Equation).pdf]

Table S1. Search equation built to include all sleep–wake disorders of the different versions of the ICSD, DSM, and ICD.

("sleep disorder*" OR "Insomnia Disorder*"OR "Sleep-related Breathing disorder*" OR "Sleep Breathing disorder*"OR "Central disorders of Hypersomnolence"OR "Circadian Rhythm Sleep Wake disorder*"OR "Circadian Rhythm disorder*"OR"Circadian disorder*" OR "Parasomnia*"OR "Sleep-related Movement disorder*"OR "Sleep Movement disorder*"OR "Obstructive Sleep Apnea" OR "Cheyne Stokes"OR "Central Sleep Apnea" OR "Obesity Hypoventilation syndrom*"OR "Congenital Central Alveolar Hypoventilation Syndrom*"OR "Late-Onset Central hypoventilation"OR "Idiopathic central alveolar hypoventilation"OR "Sleep related hypoxemia"OR "Narcolepsy"OR "Idiopathic hypersomnia"OR "Kleine Levin"OR "Insufficient Sleep syndrom*"OR "Sleep Wake Phase Disorder*"OR "Sleep Wake Rhythm Disorder*"OR "Shift Work Disorder*"OR "Jet lag disorder*"OR "parasomnia" OR “Disorders of arousal” OR "Confusional arousal"OR "Sleepwalking"OR "Sleep Terror"OR "Sleep related eating disorder*"OR"REM Sleep Behavior Disorder*"OR “Sleep Behavior Disorder*" OR "RBD" OR "Recurrent Isolated Sleep paralysis"OR "Nightmare Disorder*"OR "Exploding head syndrom*"OR "Sleep related Hallucination"OR "Sleep enuresis"OR "Restless Legs syndrom*"OR "Periodic Limb Movement"OR "Sleep related Leg Cramps"OR "Sleep related Bruxism"OR "Sleep Bruxism" OR "Sleep related rhythmic movement disorder*"OR "Sleep rhythmic movement disorder*" OR "Benign Sleep Myoclonus" OR "Propriospinal Myoclonus at Sleep onset") AND **(**(reliability OR Kappa OR agreement) AND ((Sleep-EVAL) OR (criteria AND diagnos*) OR (ICSD OR ICSD-* OR DSM OR DSM-* OR ICD OR ICD-*)) OR “field trial”**)**
