# Supplementary material for: A Systematic Review of Sleep–Wake Disorder Diagnostic Criteria Reliability Studies
Source: Biomedicines. 2022 Jul 6;10(7):1616. doi: 10.3390/biomedicines10071616 (PMC9313077; doi:10.3390/biomedicines10071616)
Supplement: Supplementary file 1 [file biomedicines-10-01616-s001.zip › Supp. Mat/Table S2 (Review).pdf]

Table S2. Description of the included studies evaluating the reliability of Sleep-Wake diagnostic criteria.

General description				*Modality of diagnostic evaluation*					*Cohen's kappa coefficient evaluation*			
First Author / Year / Country / Journal	Monocentric / Multicentric	Main category and Sleep-Wake disorders evaluated*	Classification use for diagnostic criteria	Number of clinical interviews performed with a patient	Characteristics and numbers of raters	Types of clinical interviews	Modality of diagnostic conclusion	Reliability guidelines	Modality of diagnosis comparison between raters, number of patients, and number of clinical judgement conclusions	Number of Cohen's kappa coefficients	Cohen's kappa coefficient (N = 69) and number of patients with the diagnosis	Mean Kappa Coefficient of the main Sleep-Wake categories (N = 41)
Vignatelli et al. / 2002 / Sleep / Italia	Multicentric (N = not reported) (Sleep Centers of National Association of Sleep Medicine)	Central Disorders of Hypersomnolence (Narcolepsy)	ICSD-R (1997)	1	7 resident neurologists or psychiatrists and 10 trainees' neurologists or neurophysiopathologists	Semi-structured face-to-face interview	By physician watching the videotaped based on the ICSD-R without and with a previous training	Guidelines for Reporting Reliability and Agreement Studies (GRRAS)	Comparison of 17 diagnostics on 10 videotapes (N = 170 diagnosis conclusion)	2	Kappa = 0.61 (before training), 0.95 (after training, N = 4)	Mean Kappa of Central Disorders of Hypersomnolence = 0.78
Loddo et al. / 2019 / Sleep and Breathing / Italia	Monocentric (Sleep Disorder Center of the Institute of Neurological Science of Bologna)	Parasomnia Disorders (Disorders of arousal)	ICSD-3 (2014)	3	1 neurologist resident and 2 general practitioners	Semi-structured face-to-face interview	By each physician at the end of their interview	No	Comparison of 3 diagnostics on 126 patients (N = 378 diagnosis conclusion)	3	Kappa = 0.89 (Confusional Arousal; N = 3); 0.87 (Sleepwalking; N = 2); 0.87 (Sleep terrors; N = 2)	Mean Kappa of Parasomnia Disorders = 0.88
Vignatelli et al. / 2005 / J. Neurol. / Italia	Monocentric (Sleep Disorder Center of the Institute of Neurological Science of Bologna)	Parasomnias Disorders (Disorders of Arousal; NREM Parasomnias Disorders)	ICSD-R (1997)	2 evaluations for 39 patients and 3 evaluations for 11 patients	2 general neurologists and 1 general practitioner	Semi-structured face-to-face interview	Automatically generated at the end of the interview	No	Comparison of 2 or 3 diagnostics on 50 patients (N = 111 diagnosis conclusion)	13	Kappa = Confusional Arousals: 0.74; Sleep terrors: -0.02; Sleepwalking: 0.36; Nightmares: 0.25; RBD: 0.46; Sleep paralysis: 0.69<br><br>N of positive diagnostic = [4.5 – 42] by disorder<br><br>(Not considered: Nocturnal leg cramps: 0.78; Rhythmic movement disorder:	Mean Kappa of Parasomnias Disorders = 0.41

											1.00; Sleep starts: 0.41; Sleep talking: 0.72; Primary snoring: 0.88; Sleep bruxism: 0.77; Sleep enuresis: 0.73)	
Vignatelli et al. / 2003 / J. Sleep Res. / Italia	Multicentric (N = not reported) (Sleep Centers of National Association of Sleep Medicine)	Parasomnia Disorders (REM Sleep Behavior Disorder)	ICSD-R (1997)	1	6 physicians (with no information on the background of the physicians)	Semi-structured face-to-face interview	By physician watching the videotaped based on the ICSD-R	No	Comparison of 6 diagnostics on 10 videotapes (N = 60 diagnosis conclusion)	1	Kappa = 0.65 (N = 5)	Mean Kappa of Parasomnias Disorders = 0.65
Hening et al. / 2008 / Sleep Medicine / USA	Monocentric (Hopkins Bayview Medical Center)	Sleep-Related Movement Disorders (Restless Legs Syndrome)	IRLSSG, 1995	3	1 sleep specialist and 1 non sleep specialist	Semi-structured face-to-face interview (and structured telephone interview with the HTDI)	By sleep specialists at the end of their interview (and automatically generated with HTDI)	No	Comparison of 2 diagnostics (and comparison between a physician interview based on the HTDI) (N = 124 diagnosis conclusion)	1	Kappa = 0.87 between the two physicians (and Kappa not computed between the HTDI and the physician) (N = 63)	Mean Kappa of Sleep-Related Movement Disorders = 0.87
Chung et al. / 2014 / Journal of Psychosomatic Research / China	Monocentric (University of Hong-Kong, Department of Psychiatry)	Insomnia Disorders	DSM-IV-TR (2000) + ICSD-2 (2005) + ICD-10 (1992) + DSM-5 (2013)	2	2 psychiatrists and interviewers (N not reported)	Semi-structured telephone interview (psychiatrists) and structured lay-administered telephone interview with the BIQ (interviewers)	Automatically generated (with BIQ) and by each physician at the end of their interview	No	Comparison of the clinician diagnosis with the BIQ diagnosis on 176 patients (N = 352 diagnosis conclusion)	4	Kappa = DSM-5 = 0.58; DSM-IV-TR = 0.72; ICD-10 = 0.48; ICSD-2 = 0.60 (number of patients with confirmed disorders not reported)	Mean Kappa of DSM-5 = 0.58 Mean Kappa of DSM-IV-TR = 0.72 Mean Kappa of ICD-10 = 0.48 Mean Kappa of ICSD-2 = 0.60
Edinger et al. / 1996 / Sleep /USA	Monocentric (Duke University Medical Center)	Insomnia Disorders	DSM-III-R (1987) + ICSD-1 (1990)	2	2 sleep specialists	Unstructured interviews conducted by a psychiatrist or a clinical psychologist	By 1) clinical interviews conducted by the psychiatrist and the clinical psychologist 2) and by the sleep specialist on the clinical data (and on PSG and sleep questionnaire)	No	Comparison of 2 diagnostics on 31 patients (N = 62 diagnosis conclusion)	2	Kappa = 0.71 for DSM-III-R, 0.68 for ICSD-1 (N = number of patients with confirmed disorders not reported)	Mean Kappa of for DSM-III-R = 0.71 Mean Kappa of ICSD-1 = 0.68
Kessler et al. / 2010 / Sleep / USA	Multicentric (N = not reported)	Insomnia Disorders	DSM-IV-TR (2000) + ICSD-2 (2005) + ICD-10 (1992)	2	7 psychologists with a specialization in sleep medicine and interviewers (N not reported)	Semi-structured telephone interview (psychologists) and structured lay-administered telephone interview with the BIQ (interviewers)	Automatically generated (with BIQ) and by each physician at the end of their interview	No	Comparison of the clinician diagnosis with the BIQ diagnosis on 203 patients (N = 406 diagnosis conclusion)	3	Kappa = DSM-IV-TR = 0.72; ICD-10 = 0;40; ICSD-2 = 0.42 (N = number of patients with confirmed disorders not reported)	Mean Kappa of DSM-IV-TR = 0.72 Mean Kappa of ICD-10 = 0.40 Mean Kappa of ICSD-2 = 0.42

**Edinger et al. / 2011 / Arch. Gen. Psychiatry / USA**	**Multicentric (N = 2) (Duke University Medical Center and Rush University Medical Center)**	**Insomnia Disorders**	**DSM-IV-TR (2000) + ICSD-2 (2005)**	**2**	**6 sleep specialists**	**Structured face-to-face sleep interview with the DCSID or unstructured face-to-face clinical interview**	**By each physician at the end of their interview**	**Convergent and discriminant validation by the multitrait-multimethod matrix (Campbell and Fiske)**	**Comparison of 2 diagnostics on patients interviewed with the DCSID or 2 diagnostics on patients interviewed with an unsupervised interview (Number of patients and clinical diagnosis conclusion not reported)**	**8**	**Structured interviews with the DSM-IV-TR: Kappa = 0.44 for Duke and Kappa = 0.08 for Rush; Unstructured interviews with the DSM-IV-TR: Kappa = 0.43 for Duke and Kappa = 0.14 for Rush / Structured interviews with the ICSD-2: Kappa = 0.51 for Duke and Kappa = 0.34 for Rush; Unstructured interviews with the ICSD-2: Kappa = 0.52 for Duke and Kappa = 0.12 for Rush (number of patients with confirmed disorders not reported)**	**Mean Kappa of DSM-IV-TR = 0.25 Mean Kappa of ICSD-2 = 0.74**
**Morin et al. / 1994 / Behavio. Therapy / USA**	**Monocentric (Sleep Medical Center of the Virginia Commonwealth University)**	**Insomnia Disorders**	**ICSD-1 (1990)**	**2**	**2 clinicians (with no information on their background)**	**Semi-structured face-to-face interview**	**By each clinician at the end of their interview**	**No**	**Comparison of 2 diagnostics on 20 patients (N = 40 diagnosis conclusion)**	**3**	**Kappa = Psychophysiological insomnia: 0.54; Insomnia associated with psychopathology: 0.64; Drug dependent insomnia: 0.70 (number of patients with confirmed disorders not reported)**	**Mean Kappa of Insomnia Disorders = 0.63**
**Schramm et al. / 1993 / Am. J. Psychiatry / Germany**	**Multicentric (N = 3) (Gottingen sleep laboratory, Sleep disorders center in Landeck and Central Institute of Mental Health in Mannheim)**	**6 Main Categories (Insomnia disorder, Central Disorder of Hypersomnolence, Sleep Related Breathing Disorders, Sleep Related Movement Disorders, Circadian Rhythm Sleep-Wake Disorders and Parasomnia Disorders)**	**DSM-III-R (1987)**	**2**	**5 psychologists with special knowledge of sleep medicine and 2 psychiatrists without knowledge**	**Structured face-to-face interviews (SIS-D)**	**By each clinician at the end of their interview**	**No**	**Comparison of 2 diagnostics on 68 patients (N = 136 diagnosis conclusion)**	**8**	**Kappa = All insomnias: 0.91; All hypersomnias: 0.90; All CWSD: 0.79; Sleepwalking disorder: 0.79; Primary insomnia: 0.86; Sleep-induced respiratory impairment: 0.64; Restless Leg Syndrome: 0.85; Narcolepsy: 0.88 (N = [5 – 54])**	**Mean Kappa of Insomnia Disorders = 0.88<br><br>Mean Kappa of Central hypersomnolence Disorders = 0.90<br><br>Mean Kappa of Sleep related breathing Disorders = 0.64<br><br>Mean Kappa of Sleep related movement Disorders = 0.85**

												**Mean Kappa of Circadian rhythm sleep-wake disorders = 0.79**<br><br>**Mean Kappa of Parasomnia disorders = 0.88**
**Buysse et al. / 1994 / Am. J. Psychiatry / USA**	**Multicentric (N = 5) (Montefiore Medical Center of the Albert Einstein College of Medicine, New-York; Hershey Medical Center of Pennsylvania State University; Western Psychiatric Institute and Clinic University of Pittsburgh School of Medicine; Henry Ford Hospital Detroit; Mayo Clinic, Rochester.**	**2 Main Categories (Insomnia disorder, Sleep Related Breathing Disorders)**	**DSM-IV (1994)**	**2**	**5 sleep specialists and 53 non sleep specialists**	**Unstructured face-to-face interviews**	**By each clinician at the end of their interview**	**No**	**Comparison of 2 diagnostics (between sleep specialist and non sleep specialists) on 216 patients (N = 432 diagnosis conclusion)**	**3**	**Kappa = Primary insomnia: 0.40; Sleep Related Breathing Disorders: 0.57; Insomnia related to mental disorder: 0.42 (N = [14 – 172])**	**Mean Kappa of Insomnia disorder = 0.41**<br><br>**Mean Kappa of Sleep related breathing disorders = 0.57**
**Taylor et al. / 2018 / J. of Clini. Sleep Medicine / USA**	**Multicentric (N = not reported)**	**6 Main Categories (Insomnia disorder, Central Disorder of Hypersomnolence, Sleep Related Breathing Disorders, Sleep Related Movement Disorders, Circadian Rhythm Sleep-Wake Disorders and Parasomnia Disorders)**	**DSM-5 (2013)**	**1**	**8 graduate and PhD-level evaluators with no prior training in sleep medicine (but they completed a 3-hour training led on the SCISD used)**	**Structured telephone interview (SCISD)**	**By 1) evaluator conducting the telephone interview 2) and by an evaluator based on an audio-recording**	**No**	**Comparison of 106 diagnostics obtain by the telephone interview and by the audio band evaluation (N = 212 diagnosis conclusion)**	**6**	**Kappa = Insomnia: 1.00; Hypersomnolence: 0.50; CRSWD: 0.50; Nightmare: 0.78; RLS: 0.83; OSAS: 0.73 (N = [7 – 93])**	**Mean Kappa of Insomnia Disorders = 1.00**<br><br>**Mean Kappa of Central hypersomnolence Disorders = 0.50**<br><br>**Mean Kappa of Sleep related**

												breathing Disorders = 0.73<br><br>Mean Kappa of Sleep related movement Disorders = 0.83<br><br>Mean Kappa of Circadian rhythm sleep-wake disorders = 0.50<br><br>Mean Kappa of Parasomnia disorders = 0.78
Ohayon et al. / 1999 / Sleep / USA	Multicentric (N = 2) (Sleep Disorder Center of Standford University and Regensburg University)	4 Main Categories (Insomnia disorder, Sleep Related Breathing Disorders, Central Disorder of Hypersomnolence, Parasomnia disorders)	ICSD-1 (1990)	2	9 sleep specialists and 2 non-specialists	Unstructured face-to-face interviews (sleep specialists) and structured face-to-face interviews with Sleep-EVAL (non-specialists)	Automatically generated (with Sleep-EVAL) and by sleep specialists at the end of their interview	No	Comparison of the sleep specialist's diagnosis with the Sleep-EVAL diagnosis on 105 patients (N = 210 diagnosis conclusion)	4	Kappa = OSAS: 0.94 (N = 40); Insomnia Disorders: 0.78 (N = 6); Narcolepsy: 0.66 (N = 3); RBD: 1.00 (N = 2)	Mean Kappa of Insomnia disorder = 0.78<br><br>Mean Kappa of Sleep related breathing disorders = 0.94<br><br>Central Disorder of Hypersomnolence = 0.66<br><br>Parasomnia disorders = 1.00
Merikangas et al. / 2014 / Sleep Medicine / USA	Multicentric (N = 2) (Sleep Disorder Center of Stanford University and Center of Sleep and Wake Disorders of Chevy Chase)	6 Main Categories (Insomnia disorder, Central Disorder of Hypersomnolence, Sleep related breathing disorders, Sleep related movement disorders, Circadian rhythm sleep-wake disorders and Parasomnia disorders)	ICSD-2 (2005)	2	Sleep specialists and interviewers trained for the structured interview (N not reported)	Unstructured face-to-face interviews (sleep specialists) and structured telephone interviews with DISP (non-specialists)	Automatically generated (with DISP) and by sleep specialists at the end of their interview	No	Comparison of the sleep specialist diagnosis with the DISP diagnosis on 225 patients (N = 550 diagnosis conclusion)	8	Kappa = Insomnia: 0.29 (N = 98); Hypersomnia: 0.41 (N = 69); Narcolepsy: 0.79 (N = 28); OSAS: 0.38 (N = 89); RLS: 0.20 (N = 14); RBD: 0.35 (N = 8); PLMD: 0.47 (N = 33); Delayed sleep phase disorder: 0.54 (N = 30)	Mean Kappa of Insomnia Disorders = 0.29<br><br>Mean Kappa of Central hypersomnolence Disorders = 0.41<br><br>Mean Kappa of Sleep related breathing Disorders = 0.38<br><br>Mean Kappa of Sleep related movement Disorders = 0.41<br><br>Mean Kappa of Circadian rhythm sleep-wake disorders = 0.54

												**Mean Kappa of Parasomnia disorders = 0.79**

BIQ: Brief Insomnia Questionnaire
DISP: Diagnostic Interview for Sleep Patterns and Disorders
DSM: Diagnostic and Statistical Manual of Mental Disorders
GRRAS: Guidelines for Reporting Reliability and Agreement Studies
ICSD: International Classification of Sleep Disorders
ICD: International Classification of Diseases
SCISD: Structured Clinical Interview for DSM-5 Sleep-Disorders

* Terminology relabeled according to the ICSD-3 classification.

The first group of methods (11/15 studies), designed as "test-retest reliability" methods, were to interviews a patient several times with a specific maximal time interval:

- **In green in the Table**: seven studies were based on the fact that a patient was interviewed by two interviewers (generally with a good level of expertise in sleep medicine), and reliability is calculated by comparing the Sleep-Wake disorders diagnosis done independently by different interviewers [18,24,26,28,30–32].
- **In blue in the Table**: four studies were based on the fact that a patient was interviewed independently at two different times, by a clinician and by an interviewer with a structured questionnaire. The clinician conducted an unstructured face-to-face [13,14] or semi-structured telephone interview [25,29].

The second group of methods (4/15 studies), designed as "joint interrater reliability" methods, were to apply diagnostic criteria by different clinician on the base of previous clinical data recordings:

- **In yellow in the Table**: two studies evaluated a single recording of a clinical interview between a patient and a sleep specialist (6 raters and 17 raters respectively – [33,34]), which was subsequently evaluated by several clinicians to estimate reliability.
- **In purple in the Table**: two studies were based on the fact that a patient was interviewed by an interviewer, which was subsequently evaluated by a clinician based on the recordings of the previous clinical interview [20,27].
